# Investigation on the efficiency of lentinan for injection combining cisplatin on treating malignant pleural effusion based on systematic review and meta-analysis

**DOI:** 10.1097/MD.0000000000038032

**Published:** 2024-05-24

**Authors:** Yixuan Wang, Haojia Wang, Keyan Chai, Siyu Guo, Yifei Gao, Jiaqi Li, Chuanqi Qiao, Siyun Yang, Jiying Zhou, Yifan Lin, Xiaomeng Zhang, Jiarui Wu

**Affiliations:** a Department of Clinical Chinese Pharmacy, School of Chinese Materia Medica, Beijing University of Chinese Medicine, Beijing, China.

**Keywords:** cisplatin, lentinan for injection, malignant pleural effusions, meta-analysis, systematic review, traditional Chinese medicine

## Abstract

**Background:**

Malignant pleural effusion (MPE) is a frequently observed complication in advanced malignant tumors. Clinical studies have shown that lentinan for injection (LNT) is beneficial for improving patients’ quality of life and prolonging their survival. The purpose of this meta-analysis is to evaluate the efficacy and safety of LNT combining cisplatin in the treatment of MPE.

**Methods:**

Randomized controlled trials (RCTs) of LNT combining cisplatin in the treatment of MPE were searched in 6 literature databases from the establishment time of each database by 2 researchers. According to the inclusion criteria, 2 researchers independently screened studies, assessed the risk of bias and conducted subgroup analyses for different outcome indicators according to the specific characteristics of the included literature. Analyzing the data by Revman software, and evaluating the stability of the results by Stata software.

**Results:**

A total of 52 RCTs were included. The results showed that combined use of LNT and cisplatin could improve the treatment effect, and the difference between groups was statistically significant (RR = 1.40, 95%CI: 1.33 ~ 1.46, *P* < .001). And the combined use of LNT could increase the quality of life (RR = 1.45, 95%CI: 1.35 ~ 1.56, *P* < .001). The using of LNT could significantly decrease the incidence of gastrointestinal reactions (RR = 0.86, 95%CI: 0.78 ~ 0.94, *P* < .001). Sensitivity analysis results showed that there were no qualitative changes in the indicator, and suggested the possibility of publication bias.

**Conclusions:**

Available evidence suggested the combined use of LNT and cisplatin showed better efficacy in treating MPE without increasing ADR incidence than using cisplatin alone. LNT is an ideal treatment for MPE, which has high clinical application value.

## 1. Introduction

Malignant pleural effusions (MPE) are characterized by the accumulation of fluid in the pleural space, resulting from lung cancer or other malignant tumors affecting the pleura, which is a frequently observed complication in advanced-stage malignancies.^[1]^ In the case of MPE, more than 3-quarters of patients suffer from lung cancer or breast cancer, with metastatic adenocarcinoma being the most prevalent subtype in China.^[2,3]^ Patients with MPE are often accompanied by symptoms such as chest tightness, palpitations, fever, shortness of breath, dry cough, and chest pain, which suggest a poor prognosis.^[4]^ Currently, the commonly employed clinical treatment methods for pleural effusion include therapeutic thoracentesis, chemical pleurodesis, indwelling thoracic drainage tube, indwelling thoracic catheter combined with chemical pleurodesis, systemic anti-tumor therapy, etc.^[5]^ Pleural effusion drainage combined with drug pleural perfusion is widely practiced in clinic due to its simplicity, minimal invasiveness, and reduced risk of individual complications. Cisplatin, a chemotherapeutic drug, has proven effective in managing pleural effusion. It can not only control the progression of cancer, but also induce inflammation and occlusion of pleural adhesion to control pleural effusion. However, cisplatin is highly toxic, and patients are prone to adverse reactions such as bone marrow suppression, gastrointestinal reactions, and liver function damage.

In recent years, as an important adjuvant treatment, traditional Chinese medicine has been widely used in clinical practice. Lentinan for Injection (LNT), appears as a white freeze-dried powder, is a kind of anti-tumor polysaccharide separated and purified from the Lentinula edodes, which are the second largest edible bacteria in China.^[6–8]^ *Lentinus edodes* polysaccharide is an effective active ingredient extracted from the fruiting body of high-quality *L edodes*. The active ingredient in *L edodes* is branched β - (1–3) -D-glucan. The main chain of *L edodes* is composed of β - (1–3) -linked glucose groups. Lentinan injection is an immunomodulator and is used as an adjuvant therapy for malignant tumors. LNT has been extensively studied for its pharmacological effects, and has demonstrated various beneficial properties. It has been found to enhance the immune system, possess anti-tumor, antibacterial, antiviral, and neuroprotective properties. Additionally, LNT exhibits pharmacological activities such as antidepressant, antioxidant and anti-aging.^[9,10]^ Several studies have suggested that LNT can effectively control MPE and reduce the occurrence of adverse drug reactions (ADR), such as gastrointestinal issues and hematological toxicity. It also improves the quality of life and extends the survival period of patients, particularly in older patients who may have difficulties tolerating aggressive chemotherapy. Therefore, considering the adverse effects of existing chemotherapy drugs, and the prominent advantages of lentinan injection in improving immunity, LNT combining cisplatin (DDP) is an effective treatment option for MPE.^[11]^

The current studies on LNT in the treatment of MPE are abundant, but there is a lack of evidence-based researches. This research aims to conduct a systematic review by screening out the studies related to LNT in the treatment of MPE. The research will utilize the method of meta-analysis and computer aided technology to comprehensively analyze and evaluate the collected studies, to clarify the efficacy and safety of LNT in the treatment of MPE, and provide new evidence-based evidence for rational use of drugs in MPE patients.

## 2. Methods

The procedure of the current research was conducted according to the Preferred Reporting Items for Systematic Reviews and Meta-Analyses (PRISMA) guidelines. The PRISMA checklist is shown in Guidelines Checklist.

### 2.1. Search strategy

To retrieve relevant literature, a comprehensive search strategy was carried out using the following databases: China National Knowledge Infrastructure, Wanfang, China Science and Technology Journal Database, Chinese Biomedical Literature Database (Sinomed), PubMed, and Embase databases. The search was conducted from the inception of each database until March 2, 2023, and a combination of MeSH terms and free text was used to identify randomized controlled trials (RCTs) investigating the use of LNT in the treatment of MPE. For example, the search strategy used in PubMed is outlined in Supplemental Digital Content, http://links.lww.com/MD/M580.

### 2.2. Inclusion criteria

#### 2.2.1. Types of studies

Clinical randomized controlled trials that reported the efficacy of LNT combining DDP in the treatment of MPE were required. If the “random” is mentioned in the literature, no matter in which language, publication year, or publication status.

#### 2.2.2. Types of participants

Participants, regardless of gender, age, race, etc, were all diagnosed according to a large amount of pleural effusion in chest by X-rays and B-ultrasound. The patients or family members accepted an informed consent form.

#### 2.2.3. Types of interventions

All RCTs conducted pleural effusion drainage and only used DDP as a chemotherapy drug for intrapleural injection. Based on the control group, the trial group only added intrapleural injection of LNT. To reduce the adverse reactions of patients, targeted treatment drugs such as lidocaine, dexamethasone, granisetron, lasix, promethazine, ondansetron (OD), and metoclopramide (MP) were also allowed to be used. The control group and trial group did not use other traditional Chinese medicine (TCM) and adjuvant methods, and the drug dosage and duration were not limited.

#### 2.2.4. Types of outcomes

The treatment effects: Refer to the WHO standard, Hongqian Liu group standard or Wuping Zhou group standard. It is divided into 4 curative effects of complete response (CR), Partial Response (PR), Stable Disease and Progressive Disease, as shown in Table 1. Treatment response rate = (CR cases + PR cases)/ total cases × 100%; Life quality evaluation: Karnofsky Performance Status (KPS) was used for evaluation, and effective was an increase of ≥ 10 points; The incidence of bone marrow suppression; The incidence of gastrointestinal reactions; The incidence of liver function injury; The incidence of renal injury; The incidence of fever; The incidence of chest pain.

**Table 1:** Content table of efficacy evaluation criteria.

	WHO standard	Hongqian Liu group standard	Wuping Tang group standard
**CR**	The hydrothorax disappears and persists for at least 4 wk	The hydrothorax disappeared completely and lasted for more than 4 wk	The hydrothorax decreased or ceased to increase after only 1 puncture and infusion, and the symptom remission lasted for more than 4 wk
**PR**	The hydrothorax decreased by more than 50% and lasted for more than 4 wk	The hydrothorax decreased by more than 50% and lasted for more than 4 wk	Hydrothorax decreased or ceased to increase after 2 aspirations and infusion, and symptom relief lasted for more than 4 wk
**SD**	The hydrothorax was reduced by <50% to more than 25% and maintained for more than 4 wk	Hydrothorax decreased by 25% ~ 50%, or increased by <25%	The symptoms could not be controlled or aggravated after more than 2 times of suction and injection
**PD**	The decrease in hydrothorax did not meet the above criteria or increased	Hydrothorax increased by more than 25%	

CR = complete response, PD = progressive disease, SD = stable disease.

### 2.3. Exclusion criteria

If the studies met these conditions were excluded: the studies were not consistent with the efficacy evaluation criteria or the required outcome types were not found; the studies used other TCM treatments in the trial or control groups; the full texts cannot be obtained. For duplicate studies, only those with complete data or the most recent literature were included.

### 2.4. Data extraction and quality assessment

The 2 researchers (YW and HW) independently screened studies by the NoteExpress software, and extracted data. In cases where there were differences in screening or data extraction, it was determined by discussing or seeking the opinion of a third researcher (XZ). After removing duplicate records, the researchers removed obvious incompatible documents by reading literature title and summary. The remaining documents were then read in full, and those that met the inclusion criteria were selected. The collected data was organized using Excel software. The extracted content included the first author name and publishing year, the number of participants in the trial and control groups, the gender and age distribution in both groups, the disease staging, KPS score, survival time, intervention measures, treatment details, outcome data, and so on.

The risk of bias in the studies was assessed using the risk of bias tool 2.0 (RoB2) from Cochrane to randomized clinical trials. RoB2 was also performed in pairs by 2 independent reviewers (KC and HW). If there was disagreement between the 2 evaluators about the risk of bias analyzed, a third reviewer (JW) performed the consensus. The evaluators examined the randomization process, deviations from intended interventions, missing outcome data, measurement of the outcome, and selection of the reported results. Thus, the studies were classified into low, some concerns, or high risk of bias.

### 2.5. Data analysis

The data collected from the included studies were analyzed using the Revman software 5.3.^[12]^ For binary variable, the Relative Risk (RR) was selected as the statistical index, while for continuous variables, the mean difference statistics were used. The 95% Confidence Interval (95% CI) was calculated for both types of variables. Heterogeneity testing is performed through *Q* test and *I^2^* test. When *P* > .1 and *I^2^* < 50%, use a fixed-effect model, otherwise the heterogeneity is excluded, and the random-effect model is used.^[13]^ To evaluate the stability of the results, the analysis was conducted by excluding one study at a time using Stata 14.0 software,^[14]^ which helped determine if the results were robust and not heavily influenced by any single study. Employing subgroup analysis, such as conducting analyses of treatment effects based on different evaluation criteria and analyzing life quality evaluation indicators according to medication dosage, enabled the assessment of differences in intervention effects across different subgroups. This approach enhanced the specificity of clinical guidance, allowing for more targeted and effective treatment strategies. Furthermore, a funnel plot was generated to assess publication bias. If the funnel plot showed asymmetry or if there was a lack of data points in certain areas, it indicated the presence of bias in the publication of the included studies.

## 3. Results

### 3.1. Literature search and screening results

**Figure 1.**
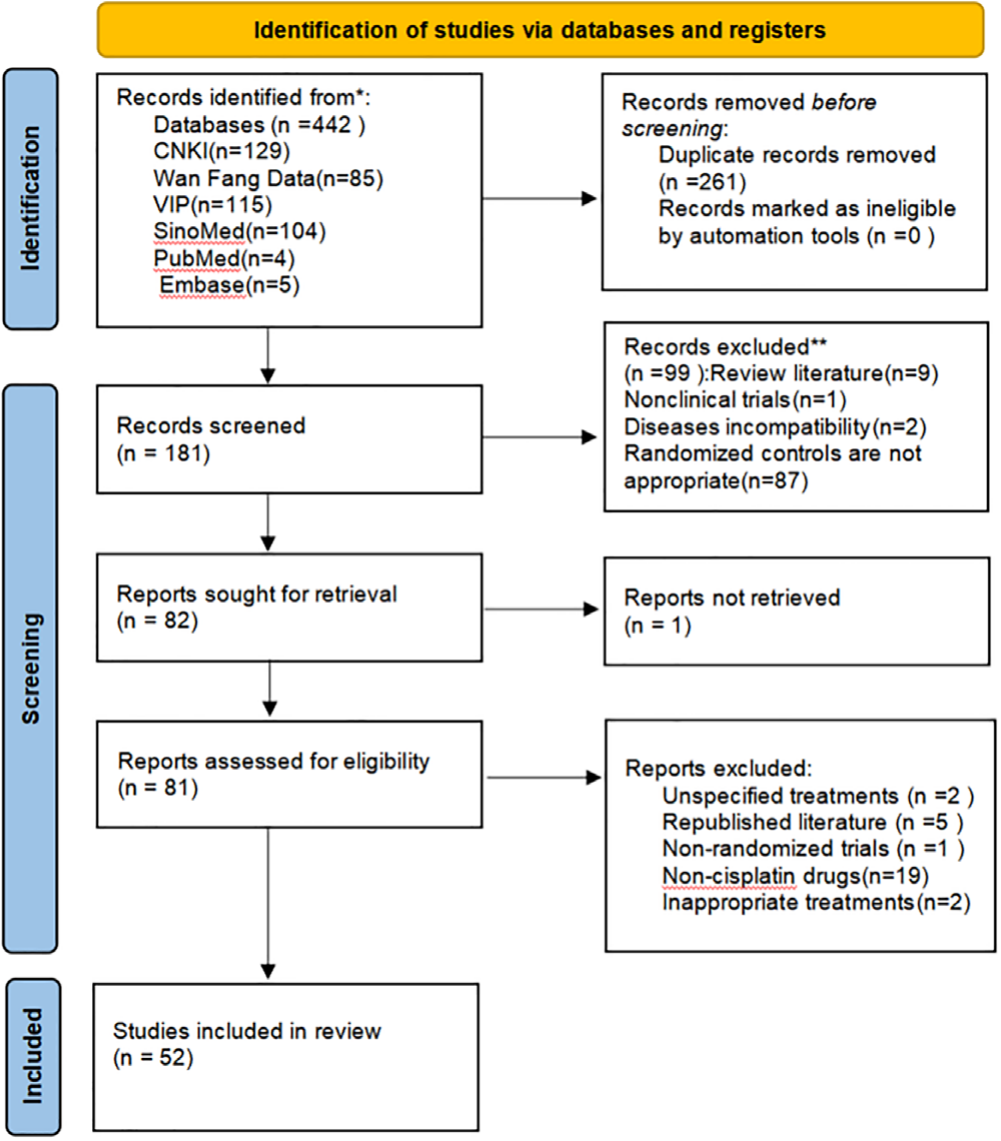
Flow chart of literature screening.

A total of 181 articles were initially detected, 82 articles were read in full, and 52 articles were finally included, all of which were published in Chinese, and the publication years were from 1996 to 2020. The literature screening process is shown in Figure 1.

### 3.2. Inclusion studies and basic characteristics

52 RCTs involved 3362 participants were included. Among these participants, 1699 were in the treatment group, while 1663 cases in the control group. The smallest sample size in the included studies was 35 cases, whereas the largest was 122 cases. More detailed characteristics are in Table 2.

**Table 2:** Characteristics of included literature characteristics.

Study ID	Cases (T/C)	Gender (M/F)	Age range (yr)	Average/median age (yr)	KPS score≥	Survival time ≥ (mo)	Control group intervention	Treatment group intervention	Other drugs (to prevent adverse reactions)	Course	Outcomes
Gang Yan 2011 (Yan and Wu, 2011)^[15]^	30/30	42/18	36~90	NR	50	NR	DDP 30mg	DDP 30mg + LNT 4mg	2%LD	4w	①②③④⑤⑥
Chunwei Xia 2011 (Xia et al, 2011)	34/34	33/35	65.2~87.5	NR	NR	3	DDP 60~90mg	DDP 60~90mg + LNT 3mg	2%LD	4w	①③④⑤⑥⑦
Junli Zhang 2012 (Zhang and Tang, 2012)^[16]^	40/40	45/35	30~72	55.3 ± 8.4/56.3 ± 9.2	NR	NR	DDP 40mg/m^2^	DDP 40mg/m^2^ + LNT 6mg	NR	1w*4	①②③④⑦⑧
Hong Wang 2018 (Wang and Ma, 2018)^[17]^	61/61	70/52	32~72	54.12 ± 10.34/52.19 ± 12.58	NR	3	DDP 30mg	DDP 30mg + LNT 4mg	DXMS + LD	4w	①③④⑦⑧
Daoming Li 2014 (Li et al, 2014)^[18]^	30/30	38/22	NR	56.1 ± 6.9/53.9 ± 7.7	50	NR	DDP 60mg	DDP 60mg + LNT 4mg	NR	therapy 3w + observation 4w	①②③④⑦⑧
Shengqin Zheng 2017 (Zheng, 2017)^[19]^	30/30	39/21	46~68	57.77 ± 6.63/58.89 ± 6.57	NR	NR	DDP 40mg	DDP 40mg + LNT 4mg	NR	NR	①③④⑦
Hongqian Liu 2018 (Liu and Lin, 2018)^[20]^	34/34	37/31	NR	62.18 ± 9.54/63.27 ± 8.96	NR	NR	DDP 40mg/m^2^	DDP 40mg/m^2^ + LNT 6mg	NR	3w	①③
Tao Jun 2018 (Tao, 2018)^[21]^	40/40	51/29	32~68	48.7 ± 6.9/48.6 ± 7.6	50	3	DDP 60mg	DDP 60mg + LNT 1mg	NR	4w	①②③④⑦⑧
Fu Jing 2006 (Fu, 2006)^[22]^	30/30	NR	33~78	51 (median)	60	3	DDP 80mg	DDP 80mg + LNT 2mg	NR	4w	①③④⑤⑥⑦⑧
Qiuyue Xing 2001 (Xing et al, 2001)^[23]^	36/34	52/18	21~71	48.6/49.5	50	NR	DDP 60~80mg	DDP 60~80mg + LNT 4mg	Dxm	NR	②④⑥⑦⑧
Wuping Tang 2010 (Tang and Liu, 2010)^[24]^	32/32	51/13	32~75	54.1	60	3	DDP 60mg	DDP 60mg + LNT 4mg	2%LD + LS + GST	1w*2~3	①②③④⑤⑥
Yong Liang 2008 (Liang, 2008)^[25]^	37/37	34/40	25~70	51 (median)	NR	NR	DDP 80mg	DDP 80mg + LNT 2mg	NR	4w	①
Jianping Tang 2020 (Tang et al, 2020)^[26]^	40/40	50/30	35~75	53.6 ± 4.8/54.3 ± 4.7	50	3	DDP 60mg	DDP 60mg + LNT 4mg	DXMS + 2%LD + LS + GST	3w*	①③④⑤⑥⑦⑧
Xiaoyu Wang 2009 (Wang et al, 2009)^[27]^	45/45	72/18	30~70	49/48 (median)	50	3	DDP 60mg	DDP 60mg + LNT 1mg	2%LD	4w	①
Sujuan Cao 2010 (Cao et al, 2010)^[28]^	40/40	55/25	40~82	NR	50	3	DDP 40mg	DDP 40mg + LNT 4mg	NR	4w	①②
Xiaolang Tan 2015 (Tan et al, 2015)^[29]^	42/42	48/36	NR	58.6/56.3	60	NR	DDP 60mg	DDP 60mg + LNT 6mg	DXMS	3w	①②③④⑦⑧
Chuanxing Wang 2012 (Wang and Zhang, 2012)^[30]^	32/32	35/29	40~70	57/60 (median)	50	3	DDP 60mg	DDP 60mg + LNT 4mg	DXMS	1w*2~3	①②③④⑦⑧
Lijun Yao 2016 (Yao et al, 2016)^[31]^	30/30	42/18	30~75	56.2 ± 16.5	60	3	DDP 40mg	DDP 40mg + LNT 6mg	NR	1w*4	①②③④⑦⑧
Zhiqiang Zhang 2010 (Zhang et al, 2010)^[32]^	39/39	61/17	26~72	52/50 (median)	NR	3	DDP 60mg	DDP 60mg + LNT 2mg	NR	1w*4	①③④⑦⑧
Yingming Wang 2017 (Wang and Zhang, 2017)^[33]^	32/30	43/19	26~69	47.0 ± 6.3/49.6 ± 6.5	NR	NR	DDP 60mg	DDP 60mg + LNT 1mg	DXMS + MP + PT	1w*1~2	①②④⑦⑧
Wei Zhou 2004 (Zhou et al, 2004)^[34]^	29/23	NR	NR	NR	40	NR	DDP 60~100mg	DDP 60~100mg + LNT 4mg	NR	4w	①②③④
Xing Feng 2009 (Feng et al, 2009)^[35]^	35/35	38/32	31.6~78.1	43 ± 2.6	NR	NR	DDP 60~90mg	DDP 60~90mg + LNT 3mg	NR	4w	①④⑤⑦⑧
Yingman Wang 2008 (Wang et al, 2008)^[36]^	40/40	49/31	NR	63.5	60	NR	DDP 50mg	DDP 50mg + LNT 4~6mg	DXMS	1w*1~3	①③④⑦
Yongmei Zhang 2008 (Zhang and Qu, 2008)^[37]^	36/26	21/41	30~70	52/50 (median)	50	3	DDP 30mg	DDP 30mg + LNT 1mg	NR	21d*2	①③④⑤⑥⑦
Hanyi Yu 2003 (Yu, 2003)^[38]^	32/32	31/33	27~69	51 (median)	NR	NR	DDP 80mg	DDP 80mg + LNT 2mg	NR	1w*4	①③④⑤⑥⑦
Zengbai Xu 2010 (Xu and Wang, 2010)^[39]^	52/52	69/35	30~72	49 (median)	NR	NR	DDP 40~80mg	DDP 40~80mg + LNT 4mg	LD + DXMS + GST + MP	1w*2	①④⑦⑧
Hongyan Xia 2016 (Xia, 2016)^[40]^	40/40	35/45	NR	58 ± 14/60 ± 12	NR	NR	DDP 60mg	DDP 60mg + LNT 2mg	2%LD + LS + GST + DXMS	1w*2~3	①②③④⑦⑧
Fu Zhou 2013 (Zhou and Yang, 2013)^[41]^	40/40	44/36	67~88	71 ± 2/72 ± 2.5	NR	3	DDP 60mg	DDP 60mg + LNT 4mg	2%LD + LS + DXMS	1w*2~3	①②③④⑤⑥
Guiping Chen 2012 (Chen and Chen, 2012)^[42]^	32/32	42/22	31~72	NR	50	3	DDP 60mg	DDP 60mg + LNT 2mg	NR	4w	①②
Xianghui Chang 2013 (Chang et al, 2013)^[43]^	40/40	33/47	NR	59 ± 16/61 ± 14	NR	NR	DDP 40~60mg	DDP 40~60mg + LNT 2mg	DXMS + 2%LD + MP/GST	7d	①②③④⑦⑧
Chunwei Xia 2012 (Xia et al, 2012)^[44]^	40/40	NR	NR	NR	NR	3	DDP 30mg	DDP 30mg + LNT 3mg	NR	1w*4	①③④⑦
Junwei Zhang 2016 (Zhang 2016)^[45]^	30/30	34/26	27~77	55.3 ± 1.2	NR	NR	DDP 40mg	DDP 40mg + LNT 6mg	NR	1w*4	①②③④⑦⑧
Wei Sun 2014 (Sun et al, 2014)^[46]^	35/35	38/32	35~70	52.5	NR	NR	DDP 40mg	DDP 40mg + LNT 10mg	NR	1w*4	①③⑦
Guiyang Feng 2013 (Feng, 2013)^[47]^	23/23	26/20	36~71	46.8	60	3	DDP 60mg	DDP 60mg + LNT 6mg	NR	1w*4	①④⑦⑧
Fenying Dong 2008 (Dong et al, 2008)^[48]^	30/29	35/24	35~76	NR	NR	NR	DDP 80mg	DDP 80mg + LNT 5mg	OD	1w*4	①②③④⑤⑥⑦⑧
Dahong Zhang 2008 (Zhang, 2008)^[49]^	30/29	33/26	38~75	60.7 (median)	50	3	DDP 60~80mg	DDP 60~80mg + LNT 5mg	NR	1w*4	①②④⑤⑥⑦⑧
Hong Qin 2000 (Qin and Xu, 2000)^[50]^	24/23	35/12	40~70	54.3/52.5	NR	3	DDP 60mg	DDP 60mg + LNT 4mg	NR	2w*2	①⑦⑧
Subing Dong 2014 (Dong, 2014)^[51]^	28/28	NR	NR	NR	NR	NR	DDP 40mg	DDP 40mg + LNT 6mg	NR	1w*4	①②③④⑤⑥⑦⑧
Jinyu Liu 2011 (Liu, 2011)^[52]^	28/26	30/24	36~70	51 (median)	60	3	DDP 60~80mg	DDP 60~80mg + LNT 4mg	DXMS + OD	1w*2~3	①②③④⑤⑥⑦⑧
Chouye Wang 2018 (Wang, 2018)^[53]^	18/18	29/7	NR	42 ± 5/43 ± 4	50	3	DDP 50mg	DDP 50mg + LNT 8mg	DXMS	1w*4	①③④⑤⑥⑦
Hua Zhang 2009 (Zhang and Qi, 2009)^[54]^	28/28	40/16	38~78	68 (median)	50	NR	DDP 40~60mg	DDP 40~60mg + LNT 4~6mg	DXMS + 2%LD	1w*2~3	①②③④
Fei Liu 2011 (Liu et al, 2011)^[55]^	28/29	37/20	31~74	56.5	60	3	DDP 40mg	DDP 40mg + LNT 6mg	NR	1w*4	①②③④⑦⑧
Hong Xu 2017 (Xu et al, 2017)^[56]^	29/29	33/25	20~80	55/56	40	3	DDP 30mg	DDP 30mg + LNT 4mg	DXMS + LD	1w*4	①②③④⑤⑥⑦⑧
Min Ye 2014 (Ye, 2014)^[57]^	31/27	31/27	36~76	56 (median)	60	3	DDP 40mg	DDP 40mg + LNT 4mg	DXMS	1w*2	①③④⑤⑥
Manchao Chai 1996 (Chai and Wang, 1996)^[58]^	18/17	21/14	27~70	48.2/50.1	NR	NR	DDP 40mg	DDP 40mg + LNT 1mg (1time/2d)	NR	4w	①③④⑤⑥⑦⑧
Lihui Geng 2005 (Geng, 2005)^[59]^	31/28	39/20	34~75	NR	50	NR	DDP 60mg	DDP 60mg + LNT 4mg	DXMS + 2%LD	1w*1~3	①③④⑦⑧
Henghu Fang 2015 (Fang et al, 2015)^[60]^	26/24	30/20	45~79	60 ± 11/63 ± 13	40	3	DDP 60mg	DDP 60mg + LNT 8mg	DXMS + LD	1w*3	①③④⑤⑦⑧
Dandan Nie 2010 (Nie et al, 2010)^[61]^	22/20	29/13	NR	55	NR	NR	DDP 60~80mg	DDP 60~80mg + LNT 2mg	GST	1w*4	①③④⑤⑥⑦⑧
Lihong Feng 2013 (Feng, 2013)^[62]^	21/21	27/15	NR	56.5/57.3	60	NR	DDP 40mg	DDP 40mg + LNT 6mg	NR	1w*2~3	①②③④⑦⑧
Hongmei Xie 2015 (Xie, 2015)^[63]^	20/20	25/15	19~80	48.9 ± 2.1/49.4 ± 2.6	NR	NR	DDP 40mg	DDP 40mg + LNT 6mg	NR	1w*4	①②③④⑦⑧
Qiong Dong 2010 (Dong et al, 2010)^[64]^	21/21	32/10	45~68	61	NR	NR	DDP 40mg	DDP 40mg + LNT 3mg	NR	1w*2	①
Zhiying Li 2007 (Li and Hu, 2007)^[65]^	28/28	35/21	41~91	64	50	2	DDP 20~40mg	DDP 20~40mg + LNT 2~4mg	DXMS + 2%LD	1w*1~2	①②③④⑦⑧

C = control group, DDP = cisplatin, F = female, GST = granisetron, KPS = Karnofsky Performance Status, LS = lasix, M = male, MP = metoclopramide, NR = not reported, OD = ondansetron, PT = promethazine, T = treatment group.

①: The treatment effect; ②: Life quality evaluation; ③: The incidence of bone marrow suppression; ④: The incidence of gastrointestinal reactions; ⑤: The incidence of liver function injury; ⑥: The incidence of renal injury; ⑦: The incidence of fever; ⑧: The incidence of chest pain.

### 3.3. The risk of bias in included studies

**Figure 2.**
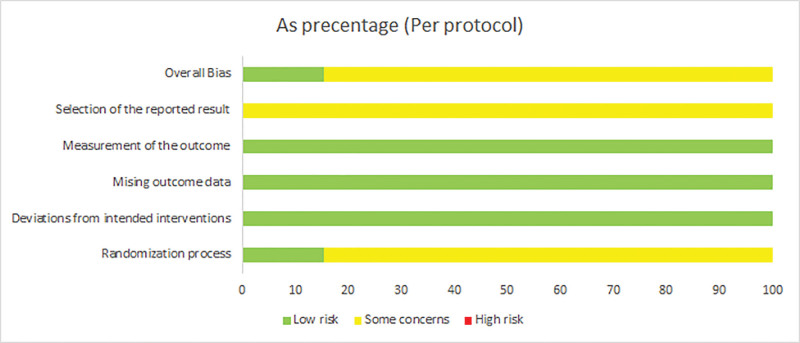
Summary of the risk of bias assessment of the included RCTs. RCTs = randomized controlled trials.

According to the RoB 2.0 criteria, among the 52 included RCTs,8 studies^[20,24–26,38,43,46,66]^ were judged as low risk (15.4%) of bias during randomization, the remaining 44 RCTs did not provide sufficient information on the generation of randomized process (84.6%). All studies were conducted according to the established intervention allocation, and there were no deviations, which was identified as low risk. All the 52 studies had complete outcome data (100%), and the outcome data were objective. According to the assessment of the bias of outcome measurement, 15.4% of the included literature were at low risk, 84.6% were some concerns, and the bias of selective reporting results was some concerns. Specific risks of bias are shown in Figure 2.

### 3.4. Results of meta-analysis

#### 3.4.1. The treatment effects

A total of 51 RCTs reported the outcome of treatment effect, involving 3292 participants. According to the different evaluation standards, the study was divided into 3 subgroups: WHO standard (47 RCTs),^[15–19,21,22,25,26,28–61,64–67]^ Hongqian Liu group standard,^[20,29,62]^ and Wuping Zhou group standard.^[24]^

In the WHO standard subgroup, 47 RCTs showed low heterogeneity (*P* = .88, *I^2^* = 0%), and a fixed-effect model was used. The result demonstrated that the treatment effect of using LNT in combination with DDP was statistically significant compared to DDP alone (RR = 1.39, 95%CI: 1.33 ~ 1.46, *P* < .001). In the Hongqian Liu group standard subgroup, the analysis of 3 RCTs’ heterogeneity was low (*P* = .65, *I^2^* = 0%), and a fixed-effect model was used. The result indicated that the treatment effect of using LNT for injection in combination with DDP was significantly higher than DDP alone (RR = 1.44, 95%CI: 1.16 ~ 1.79, *P* = .001). There was 1 RCT in Wuping Zhou group standard subgroup. The result showed that the treatment effect of LNT was higher than DDP alone (RR = 1.45, 95%CI: 1.08 ~ 1.94).

**Figure 3.**
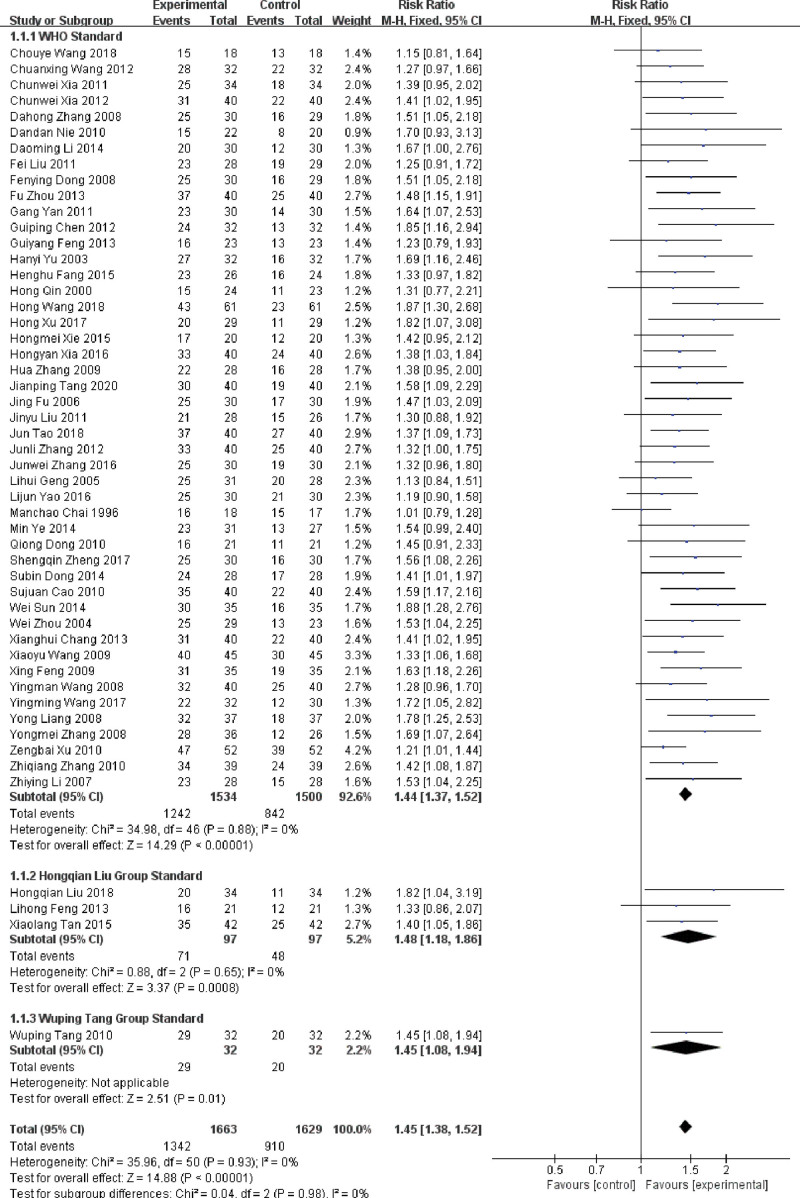
Forest plot of the treatment effect LNT in the treatment of MPE. LNT = lentinan for injection, MPE = malignant pleural effusions.

There was no significant heterogeneity between the 3 subgroups (*P* = .93, *I^2^* = 0%), so they were merged. The overall result showed that treatment effect of using DDP combined with LNT in the treatment of MPE was higher than that of DDP alone, and the difference between groups was statistically significant (RR = 1.40, 95%CI: 1.33 ~ 1.46, *P* < .001), which was shown in Figure 3.

#### 3.4.2. Sensitivity analysis in the treatment effects

**Figure 4.**
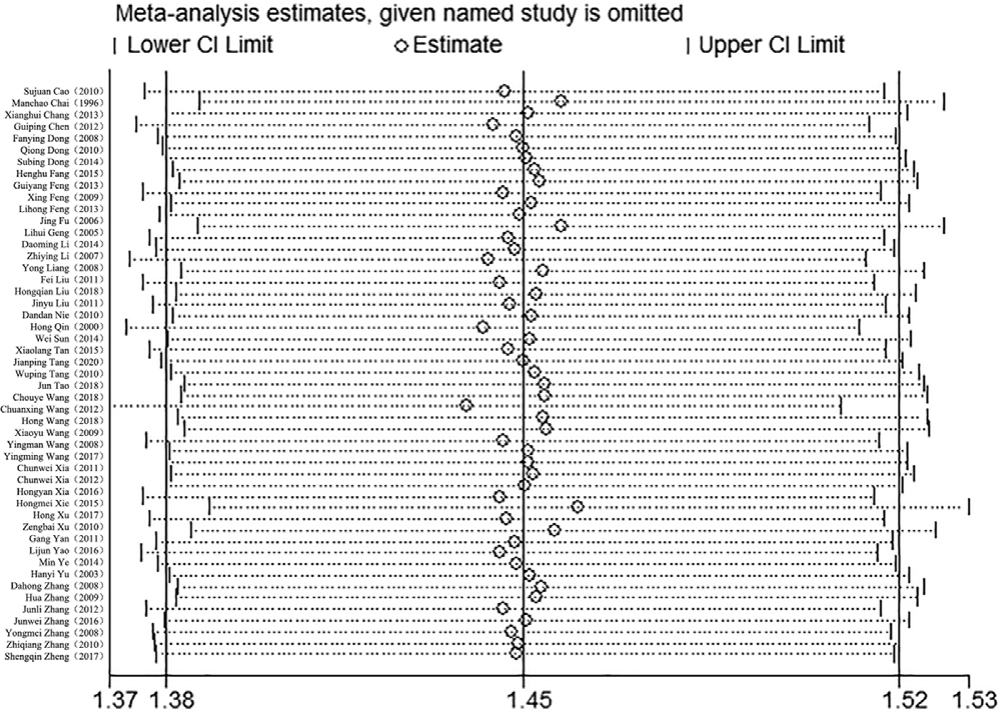
Distribution of sensitivity to the treatment effect of LNT in the treatment of MPE. LNT = lentinan for injection, MPE = malignant pleural effusions.

**Figure 5.**
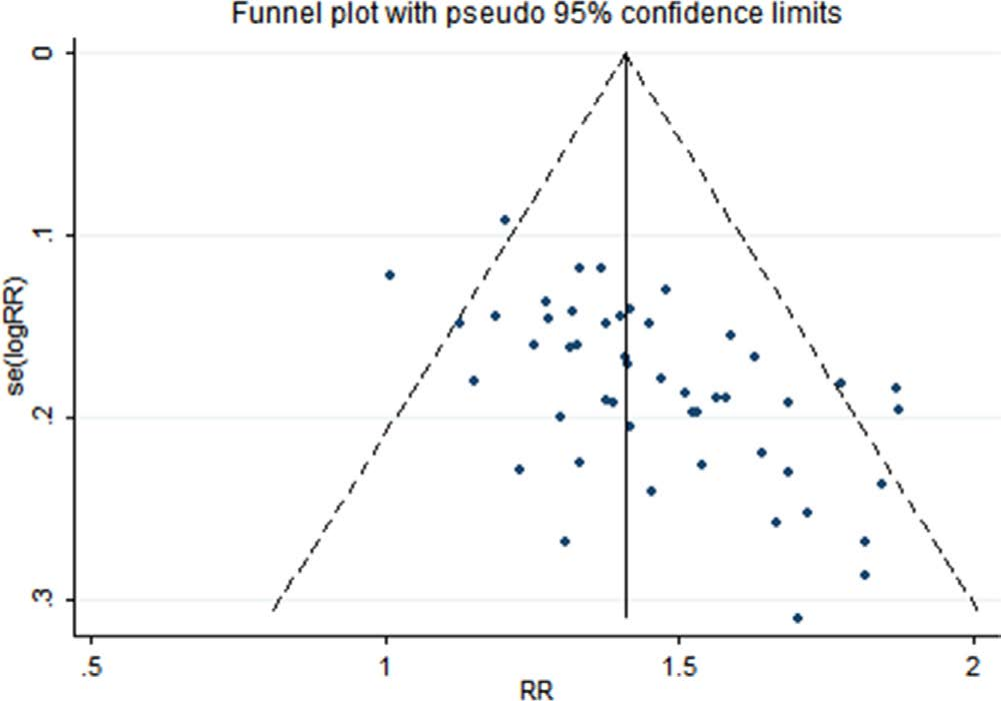
Funnel plot of the treatment effect of LNT in the treatment of MPE. LNT = lentinan for injection, MPE = malignant pleural effusions.

Sensitivity analysis results showed that there were no qualitative changes in the indicator, which indicated the treatment effect results remained consistent and stable (Fig. 4). Additionally, publication bias was assessed to determine if there was any bias in the selection and publication of studies. Figure 5 displays the distribution of points on both sides, indicating some asymmetry and partial leaning. This suggests the possibility of publication bias, where studies with positive or significant results are more likely to be published, while those with negative or non-significant results may be less likely to be published.

#### 3.4.3. Life quality evaluation

**Figure 6.**
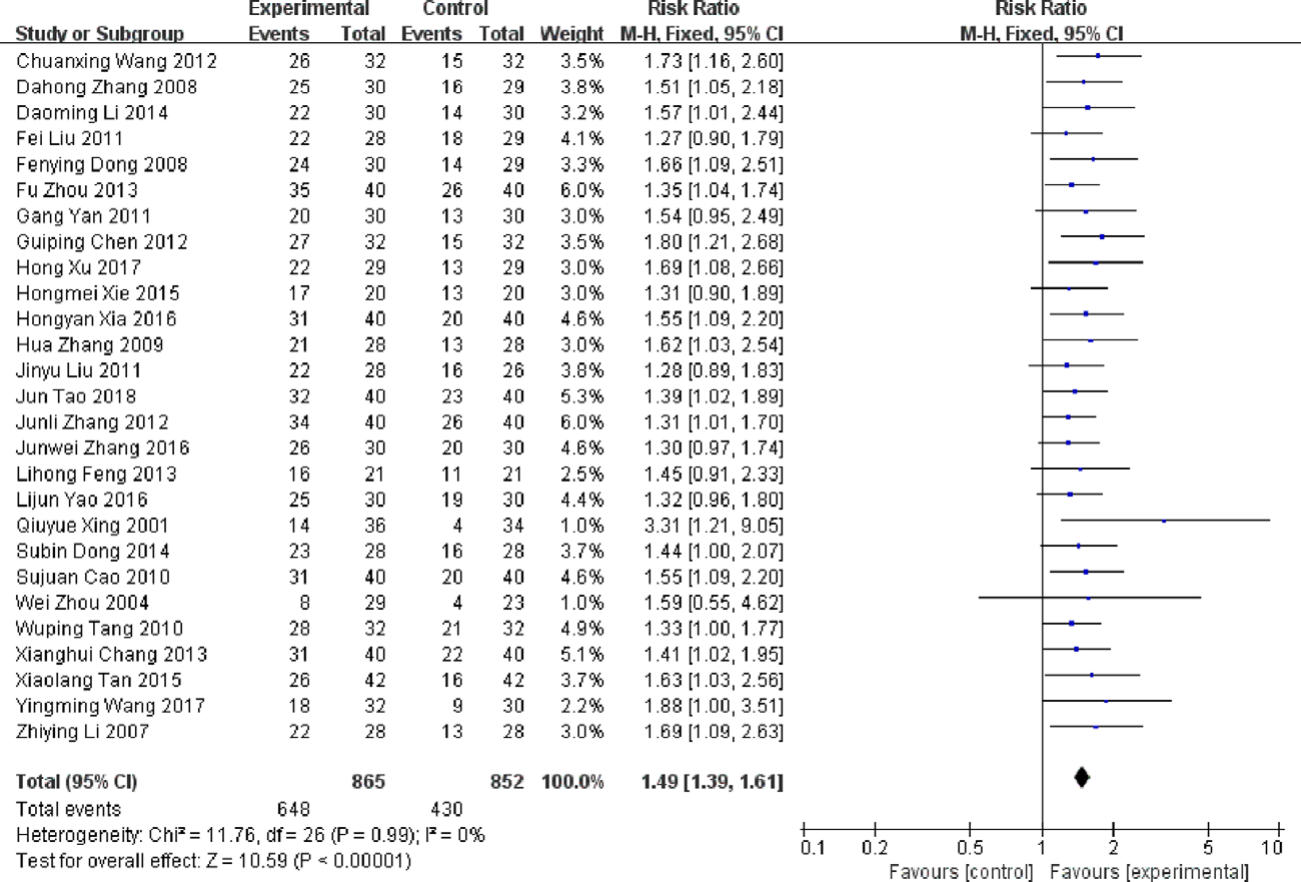
Forest plot of life quality evaluation in MPE treated with LNT. LNT = lentinan for injection, MPE = malignant pleural effusions.

Among the 52 RCTs included, there were 27 RCTs reported the life quality evaluation.^[15,16,18,21,23,24,28–31,33,34,40–43,45,48,49,51,52,54–56,62,63,65]^ After analysis, heterogeneity was low (*P* = .99, *I^2^* = 0%), and a fixed-effect model was used. The result showed that based on DDP, the use of LNT increased the quality of life. The difference between groups was statistically significant (RR = 1.45, 95%CI: 1.35 ~ 1.56, *P* < .001) (Fig. 6).

The 27 RCTs were further divided into 3 subgroups according to the doses of cisplatin, which categorized as follows: Doses below 40mg (including 40mg), Doses of 40mg to 60mg (including 60mg), and doses above 60mg. Table 3 provides a tabulated summary of the *RR* values for each subgroup. The results indicated that the subgroup of doses above 60mg had the highest *RR* value, doses of 40mg to 60mg had a slightly lower *RR* value, while the subgroup with doses below 40mg had the lowest. These findings suggested that higher doses of cisplatin may lead to a stronger treatment effect compared to lower doses.

**Table 3:** Results for life quality evaluation subgroup analysis of LNT in the treatment of MPE.

Subgroups	RCTs	Participants	Heterogeneity		MD (95% CI)	*P*
			*P*	*I * ^2^		
Doses below 40mg (including 40mg)	11	649	.98	0%	1.39 (1.25, 1.55)	<.001
Doses of 40mg to 60mg (including 60mg)	11	774	.95	0%	1.49 (1.34, 1.66)	<.001
Doses above 60mg	5	294	.44	0%	1.51 (1.23, 1.87)	<.001
Total	27	1717	.99	0%	1.45 (1.35, 1.56)	<.001

MD = mean difference, MPE = malignant pleural effusions, RCTs = randomized controlled trials.

#### 3.4.4. The incidence of bone marrow suppression

**Figure 7.**
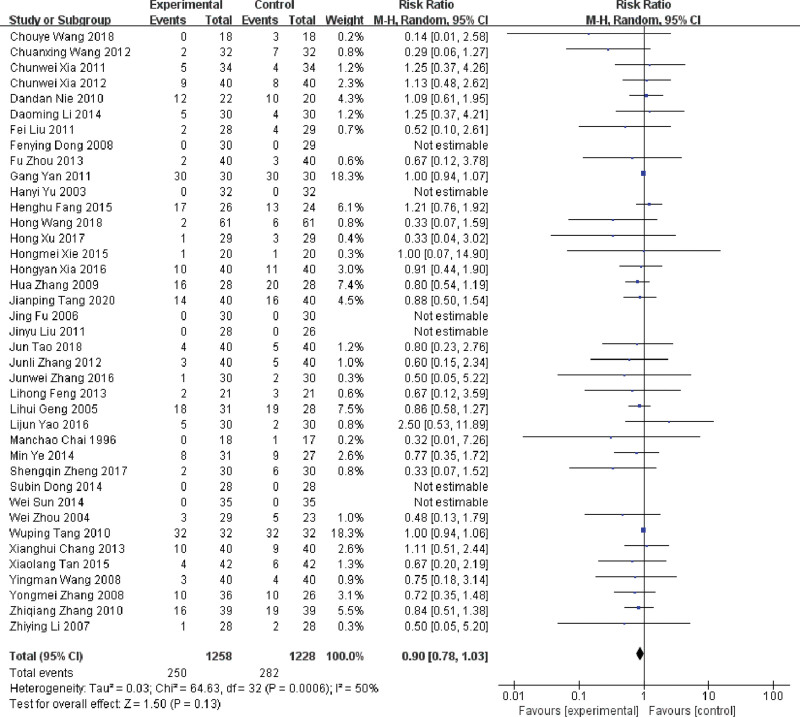
Forest plot of the incidence of bone marrow suppression in MPE treated with LNT. LNT = lentinan for injection, MPE = malignant pleural effusions.

Among the 52 included RCTs, there were 39 RCTs reported the incidence of bone marrow suppression rate.^[15–19,21,22,24,26,29–32,34,36–38,40,41,43–46,48,51–63,65,67]^ After analysis, heterogeneity was high (*P* < .001, *I^2^* = 50%), and a random-effect model was used. The result indicated that the use of LNT did not show a statistically significant difference in reducing the incidence of bone marrow suppression when compared to DDP (RR = 0.90, 95%CI: 0.78 ~ 1.03, *P* = .13), as shown in Figure 7.

#### 3.4.5. The incidence of gastrointestinal reactions

**Figure 8.**
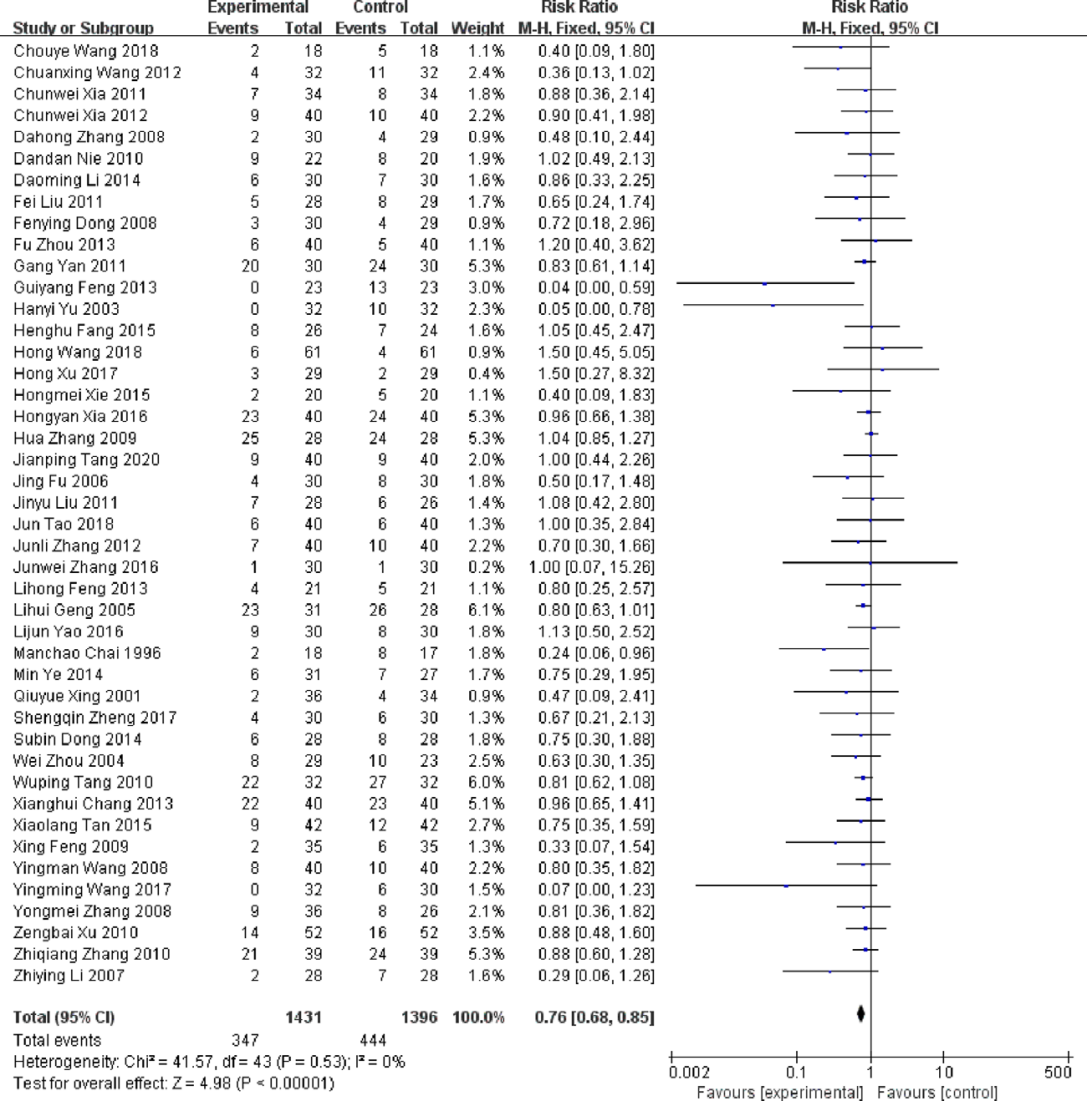
Forest plot of the incidence of gastrointestinal reactions of MPE treated with LNT. LNT = lentinan for injection, MPE = malignant pleural effusions.

Among the 52 included RCTs, there were 44 RCTs reported the incidence of gastrointestinal reactions.^[15–19,21–24,26,29–41,43–45,47–49,51–63,65,67]^ The heterogeneity between 44 RCTs was low (*P* = .53, *I^2^* = 0%), the fixed-effect model result indicated that the incidence of gastrointestinal reactions was reduced by using LNT, which difference between groups was statistically significant (RR = 0.86, 95%CI: 0.78 ~ 0.94, *P* < .001) (Fig. 8).

#### 3.4.6. The incidence of liver function injury

**Figure 9.**
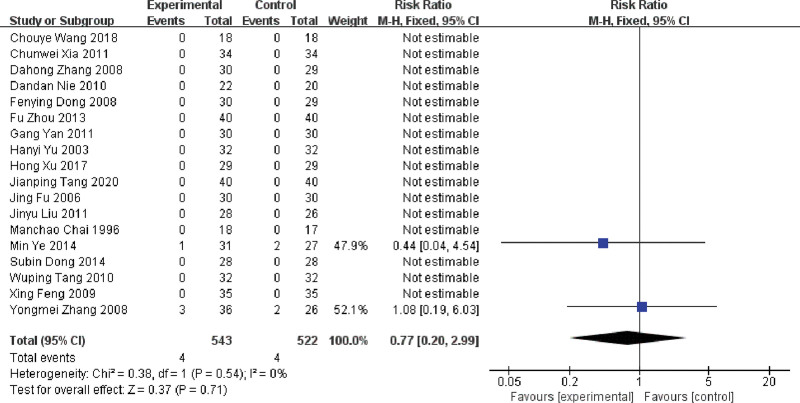
Forest plot of the incidence of liver function injury in MPE treated with LNT. LNT = lentinan for injection, MPE = malignant pleural effusions.

18 RCTs reported the incidence of liver function injury, 2 of which had a liver injury,^[37,57]^ and the remains were normal. As shown in the Figure 9, a fixed-effect model (*P* = .54, *I^2^* = 0%) was conducted. The result showed that the combined use of LNT was not significant in reducing the incidence of liver injury (RR = 0.79, 95%CI: 0.20 ~ 3.15, *P* = .74).

#### 3.4.7. The incidence of renal injury

18 RCTs reported the incidence of renal injury, in which none of the participants suffered renal injury.

#### 3.4.8. The incidence of fever

**Figure 10.**
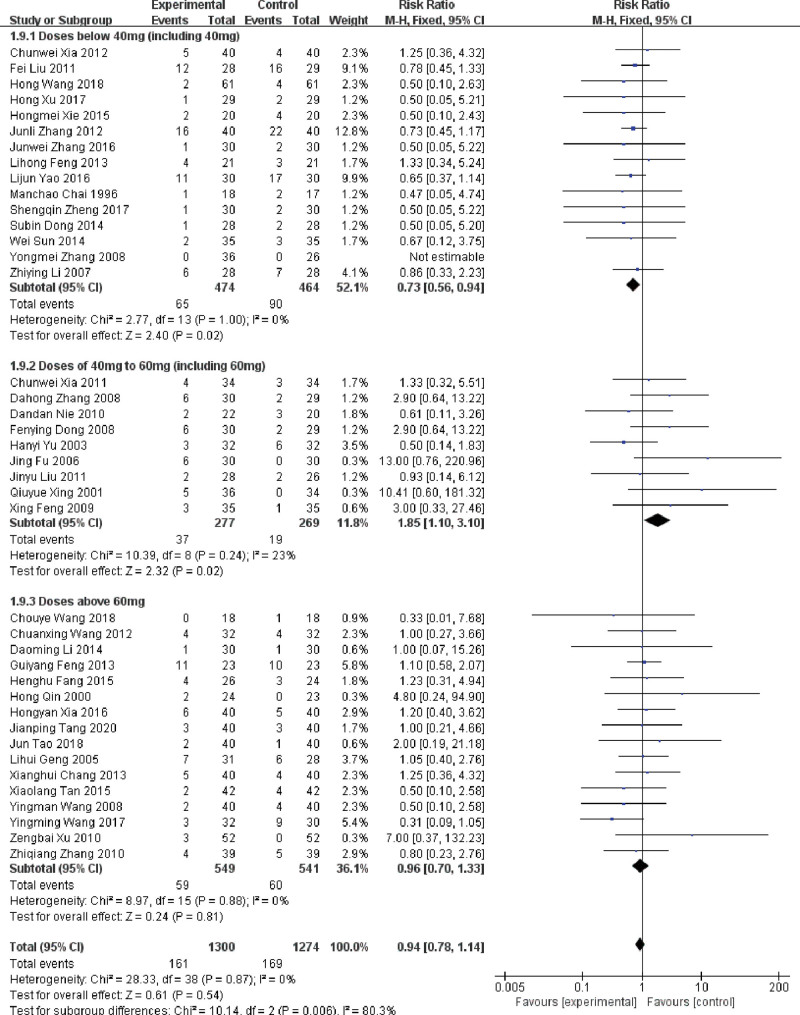
Forest plot of subgroup analysis of the incidence of fever in MPE treated with LNT. LNT = lentinan for injection, MPE = malignant pleural effusions.

There were 40 RCTs reported the incidence of fever.^[16–19,21–23,26,29–33,35–40,43–53,55,56,58–63,65,67]^ After heterogeneity test (*P* = .87, *I^2^* = 0%), a fixed-effect model was conducted, which indicated that the difference of the incidence of fever between the trial and control group was not statistically significant (RR = 0.87, 95%CI: 0.72 ~ 1.05, *P* = .15). According to the dosage of cisplatin, the 40 RCTs could be further divided into 3 groups, including the subgroup of dosage of 40mg (including 40 mg) or less, 40 ~ 60mg (including) and dosage of 60mg. The results of the combined subgroups showed that different dosages of cisplatin may affect the incidence of fever, which means the higher the dose of DDP, the higher the incidence of fever and the smaller the intervention effect of LNT (Fig. 10).

#### 3.4.9. The incidence of chest pain

**Figure 11.**
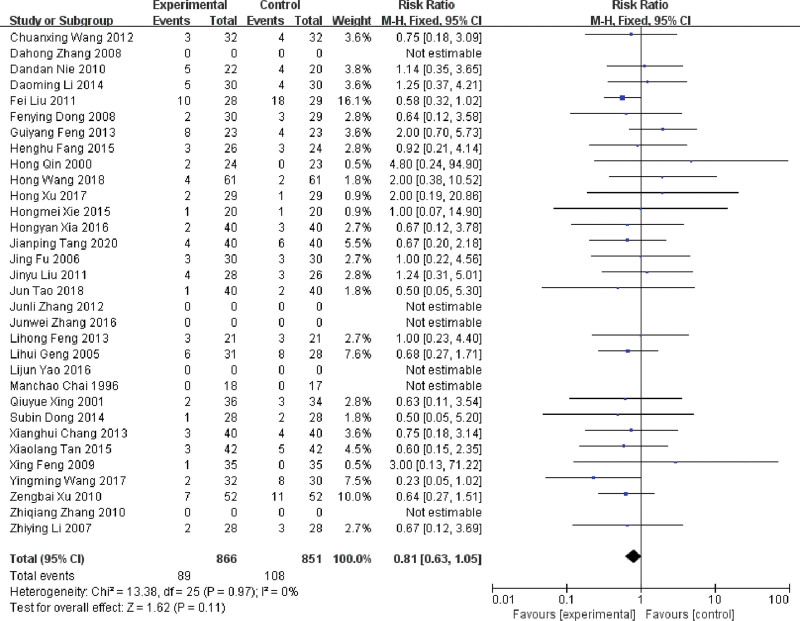
Forest plot of the incidence of chest pain in MPE treated with LNT. LNT = lentinan for injection, MPE = malignant pleural effusions.

32 RCTs compared the incidence of chest pain.^[16–18,21–23,26,29–33,35,39,40,43,45,47–52,55,56,58–63,65]^ After heterogeneity test (*P* = .97, *I^2^* = 0%), a fixed-effect model showed that the difference of the incidence of chest pain between the trial and control group was not statistically significant (RR = 0.79, 95%CI: 0.61 ~ 1.03, *P* = .08), as shown in Figure 11.

## 4. Discussion

In the studies, a comprehensive meta-analysis of 52 RCTs was conducted, which involved a total of 3, 362 participants. The meta-analysis revealed that the combination of LNT with DDP resulted in a significantly higher treatment effect for MPE compared to DDP alone. At the same time, the use of LNT was associated with improved quality of life. In terms of ADR, the combined use of LNT could significantly decrease the incidence of gastrointestinal reactions, and relieve the incidence of bone marrow suppression, liver function injury, fever, and chest pain to a certain extent. Overall, the combined use of LNT with DDP showed good efficacy in the treatment of MPE without increasing the ADR incidence. Therefore, LNT is an ideal treatment for MPE, which has high clinical application value.

The anti-tumor and autoimmune functions of LNT have been demonstrated in clinical pharmacology experiments. LNT acts as a biological response modulator, meaning that it does not directly target tumor cells but acts on host mediation.^[27]^ By doing so, LNT can improve and activate the host defense mechanism, assisting in the production of anti-tumor effects. MPE is characterized by the accumulation of fluid in the pleural space due to the presence of tumor cells. By enhancing the host defense mechanism, LNT can potentially inhibit tumor growth and metastasis, leading to a reduction in the accumulation of fluid in the pleural space. This can alleviate symptoms such as dyspnea, chest pain, and coughing, improving the patient quality of life. LNT can stimulate the body T cells, activate macrophages, and play a natural killing role in the body-dependent macrophages and natural killer cell (NK) cytotoxic effect, and finally achieve the purpose of anti-tumor. The combination of LNT and chemotherapy drugs can play a certain synergistic effect, and has a protective and regulatory effect on the immune function of patients after chemotherapy. Activation of the exudate immune cells in the chest and abdomen, and then indirectly kill or inhibit tumor cells.^[68]^ Studies have confirmed that local administration of LNT can promote local chemical reactions and form chemical pleurisy. At the same time, it can also stimulate pleural adhesion and fixation, thicken the pleural cavity, block the pleural cavity, reduce the permeability of tumor blood vessels and hinder the formation of tumor blood vessels, and enhance the interstitial reaction of fibrous hyperplasia in tumor tissue. It can activate the function of immune cells in the thoracic cavity, enhance the induction of tumor-specific killer T cells, enhance the body immunity, and reduce its side effects.

This study has the following innovation points worth highlighting. Firstly, it only included studies in which the generation of the randomized method was scientifically sound which enhanced the reliability and validity of the evidence-based findings for the clinical use of LNT combined with DDP in MPE. Secondly, the research indicators used in this study are comprehensive. In addition to the analysis of treatment effects and life quality, the study also paid attention to various outcome indicators such as the ADR incidence, including bone marrow suppression, gastrointestinal reactions, liver function injury, renal injury, fever, and chest pain. By considering multiple outcome measures, the study provided a more holistic understanding of the impact of LNT combined with DDP in the treatment of MPE. Conversely, there were some limitations in the study: the included studies were all Chinese articles, which may be the geographical limitations of LNT in clinical use. According to the trend of point distribution in the publication bias chart, there is publication bias in this study. Studies with smaller sample sizes were more likely to show a better effect of the trial group, and the RR value of the study result was larger. It probably stems from the selective reporting of clinical study results. From a practical perspective, more positive results are more likely to be published, and the negative results are relatively difficult to obtain. Therefore, we suggest that clinical researchers develop more rigorous and scientific clinical research protocols, conduct multi-center, large-sample double-blind experiments, and produce more convincing research results.

## 5. Conclusion

In summary, the study uses the meta-analysis method to comprehensively analyze and evaluate the efficacy and safety of LNT combining DDP in the treatment of MPE, to provide a new evidence-based medical basis for the rational use of MPE patients. If the future appears more large-sample, multi-center, prospective, randomized, double-blind studies, the certainty of the evidence will be higher.

## Author contributions

**Conceptualization:** Xiaomeng Zhang.

**Data curation:** Haojia Wang, Keyan Chai.

**Funding acquisition:** Siyu Guo, Chuanqi Qiao.

**Methodology:** Yifei Gao.

**Project administration:** Jiaqi Li, Jiarui Wu.

**Resources:** Siyun Yang, Jiying Zhou, Yifan Lin.

**Software:** Keyan Chai.

**Supervision:** Xiaomeng Zhang, Jiarui Wu.

**Writing – original draft:** Yixuan Wang.

**Writing – review & editing:** Haojia Wang.

## Supplementary Material


