# Supplementary material for: Investigation on the efficiency of lentinan for injection combining cisplatin on treating malignant pleural effusion based on systematic review and meta-analysis
Source: Medicine (Baltimore). 2024 May 24;103(21):e38032. doi: 10.1097/MD.0000000000038032 (PMC11124763; doi:10.1097/MD.0000000000038032)
Supplement: Supplementary file 1 [file medi-103-e38032-s001.docx]

**PubMed search strategy**

| **NO.** | **Term** |  |
| --- | --- | --- |
| #1 | Malignant Pleural Effusion | [Mesh] |
| #2 | Effusion, Malignant Pleural | [Title/Abstract] |
| #3 | Effusions, Malignant Pleural | [Title/Abstract] |
| #4 | Malignant Pleural Effusions | [Title/Abstract] |
| #5 | Pleural Effusions, Malignant | [Title/Abstract] |
| #6 | MPE | [Title/Abstract] |
| #7 | #1 OR #2 OR #3 OR #4 OR #5 OR #6 |  |
| #8 | lentinan | [All Fields] |
| #9 | LNT | [All Fields] |
| #10 | Lentinan for injection | [All Fields] |
| #11 | lentinan injection | [All Fields] |
| #12 | Injectable lentinan | [All Fields] |
| #13 | Tian Di Xin | [All Fields] |
| #14 | #8 OR #9 OR #10 OR #11 OR #12 OR #13 |  |
| #15 | #7 AND #14 |  |
